# Correlation of Wnt5a expression with histopathological grade/stage in urothelial carcinoma of the bladder

**DOI:** 10.1186/1746-1596-8-139

**Published:** 2013-08-15

**Authors:** Ramiro Malgor, Seth Crouser, Danielle Greco, Colin Brockett, Karen Coschigano, Masato Nakazawa, Scott Jenkinson

**Affiliations:** 1 Department of Biomedical Sciences, Heritage College of Osteopathic Medicine, Ohio University, Athens, OH 45701-2979, USA; 2 Office of Research and Grants, Heritage College of Osteopathic Medicine, Ohio University, Athens, OH, USA; 3 Department of Specialty Medicine, Heritage College of Osteopathic Medicine, Ohio University, Athens, OH, USA

**Keywords:** Bladder cancer, Wnt5a, Urothelial carcinoma, Cell migration

## Abstract

**Background:**

Bladder cancer, including urothelial carcinoma (UC), is the most common malignancy of the urinary tract and the fourth most frequent cancer overall in men. Wnt5a, a member of the Wnt family of proteins, has been shown to have contradictory roles in the pathogenesis of many cancers, acting either as tumor suppressor or tumor promoter. The objective of this study was to investigate the expression and role of Wnt5a in the pathogenesis of UC and suggest possible clinical applications for diagnosis, prognosis and treatment.

**Methods:**

We characterized the expression of Wnt5a in 33 human UC samples using immunohistochemistry. The samples were obtained via transurethral resection, immediately fixed in formalin and then embedded in paraffin. The correlation between Wnt5a immunoreactivity, histological grade, and pathological stage of the tumor was analyzed. The expression of Wnt5a mRNA as well as the effect of Wnt5a on cell migration was also evaluated in two UC cell lines, T24 and J82, and a normal urothelial cell line.

**Results:**

Our immunohistochemical results revealed that Wnt5a staining intensity correlated positively with the histological grade and pathological stage of the UC. Wnt5a mRNA expression differed widely in the three urothelial cell lines, with high levels in one carcinoma cell line and low levels in the other cell line in comparison to the normal urothelial cell line. Migration increased in both UC cell lines in response to Wnt5a treatment.

**Conclusions:**

Our results show that the Wnt5a pathway may play a role in the pathogenesis of UC and suggest that Wnt5a may serve as an additional, complementary diagnostic/prognostic marker for UC.

**Virtual slide:**

http://www.diagnosticpathology.diagnomx.eu/vs/1952312091979566

## Background

Urinary bladder cancer is the fourth most frequent cancer overall in men in developed countries [1] and the most common malignancy of the urinary tract [2,3]. Worldwide, 297,338 new cases and 112,255 deaths due to bladder cancer in males were reported in 2008 [3]. In the United States, it is estimated that there will be 54,610 new cases of bladder cancer and 10,820 deaths in 2013 [4]. Approximately 95% of the tumors arising in the bladder originate in the urothelium, resulting in urothelial carcinoma (UC). Squamous cell carcinoma and adenocarcinoma are the other major forms of malignant tumors in the bladder.

Two important biological features of UC are its recurrence and its progression from superficial to invasive tumors. Approximately 50-65% of patients with noninvasive UC develop a recurrent tumor within 5 years after the first treatment [3]. A majority of tumors at pTa stage are low grade and less than 5% of the tumors in this group will progress to a higher stage. In contrast, most of the tumors at pT1 pathological stage, which include tumors with invasion into the lamina propria, are high grade; 30-50% of the tumors in this group will progress to a higher stage [1,2]. These features make UC challenging in clinical practice in relation to diagnosis, prognosis, treatment, or follow-up for each individual patient.

Wnt proteins are a group of secreted glycoproteins that play critical physiological roles during embryonic development, regulating cell proliferation, morphology, motility and fate [5]. Aberrant Wnt signaling has been implicated in pathological conditions such as cancer and inflammatory diseases [6-10]. The canonical or β-catenin dependent signaling pathway is well recognized pathway in the Wnt family and is involved in normal cell growth and differentiation as well as in cancer. In the context of bladder cancers the hypermethylation of Wnt antagonist genes such as Wnt inhibitory factor 1 (WIF1), mutations in adenomatous polyposis coli (APC) and aberrant expression of β-catenin have been reported to be associated with increased aggressiveness and poor prognosis [11-15]. Recently, epigenetic deregulation of Wnt pathway inhibitors has been implicated in abnormal activation of the canonical Wnt signaling pathway in bladder tumors [16]. All of these findings demonstrated the probable role of the canonical Wnt signaling pathway in the pathogenesis of UC of the bladder.

Wnt5a is a member of the Wnt family that belongs to a non- canonical/non- transforming signaling pathway. The non-canonical signaling involves numerous cellular pathways, all of them β-catenin-independent; however, based on the specific Wnt receptors present, Wnt5a can inhibit or promote β-catenin dependent signaling, which results in contradictory roles of Wnt5a in cancer [17]. Wnt5a has been described as a tumor promoter and a tumor suppressor for different malignancies [6,17]. It has been implicated in cancer progression as a potent enhancer of cell motility and invasiveness for malignant melanoma [18]*.* Up-regulation of Wnt5a has been observed in lung, stomach, colon, breast, pancreas and prostate cancer as well [7,19,20]. The opposite function for Wnt5a, as a tumor suppressor, has been described in hematopoietic, brain, thyroid and colorectal cancers [21-23]. Specifically, Wnt5a has been shown to act as a tumor suppressor for colorectal carcinoma by antagonizing canonical Wnt/β-catenin signaling [23].

The role of Wnt5a in the pathogenesis/progression of UC has not been fully elucidated [14,15]. Olson et al. reported the role of Wnt5a as a tumor suppressor gene in UC because ectopic expression of human Wnt5a in a UC cell line lacking the chromosomal region where Wnt5a resides abolished the cell’s tumorigenic capacity [24]. Despite efforts to clarify the role of the non-canonical Wnt signaling pathway, and Wnt5a in particular, in the pathogenesis of UC, much is still unknown.

The aim of this study was to investigate the expression of Wnt5a protein in human UC. To accomplish this, we examined the expression of Wnt5a by immunohistochemistry (IHC) in 33 formalin-fixed, paraffin-embedded (FFPE) human UC samples. We found a significant positive correlation between Wnt5a expression and the histological grade and pathological stage of the tumor. Using *in vitro* methods, we also found that Wnt5a may be involved in the migration of malignant UC cells, which could have implications regarding the invasiveness of the tumor.

## Materials and methods

### Human urothelial carcinoma tissue specimens

Samples from 33 patients collected at the time of diagnostic/therapeutic transurethral resection and diagnosed as urothelial carcinoma of the bladder were included in this study. FFPE tissue blocks were archived at the University Medical Associates - Pathology Department (UMA-Pathology lab, Athens, OH). Ethical approval for the study was obtained from the Ohio University Institutional Review Board (IRB 07E112).

The diagnosis and classification of the 33 samples were performed according to the WHO/ISUP consensus classification system and the American College of Surgeon’s Cancer Program Standards. Hematoxylin and eosin (H&E) stains were used for diagnosis and staging of the tumors. Pathological stage was determined by the degree of invasion into the lamina propria. Histological grade was defined based on architecture, polarity, thickness, and cohesiveness, as well as cytologic features including pleomorphism, chromasia, presence of nucleoli, mitosis and umbrella cells.

### Immunohistochemistry

FFPE tissue specimens were immunostained for Wnt5a using a Wnt5a polyclonal antibody. For each case, two consecutive 4 μm sections were mounted onto Superfrost glass slides. Sections were deparaffinized followed by antigen retrieval using 10 mM citrate buffer, pH 6.0, at 90°C for 30 minutes. Endogenous peroxidase activity was blocked with 3% H_2_O_2_ in phosphate buffered saline (PBS). Endogenous biotin and avidin were blocked using the Streptavidin/Biotin Blocking Kit (Vector Laboratories, Inc., Burlingame, CA). Rabbit polyclonal antibody against human Wnt5a (Santa Cruz Biotechnology, Santa Cruz, CA) was applied to one of the two sections at 4 μg/ml diluted in 1% bovine serum albumin (BSA), in PBS. As an isotype control, normal rabbit IgG (Invitrogen, Grand Island, NY) was applied to the other section at the same concentration. Slides were incubated overnight at 4°C in a humidified chamber. A peroxidase-based visualization kit, Universal LSAB™ + Kit/HRP, Rabbit/Mouse/Goat, was subsequently used according to the manufacturer’s protocol (Dako North America, Inc., Carpinteria, CA). Briefly, after three washes, the slides were incubated with the biotinylated secondary antibody for 20 minutes, washed three times, incubated with streptavidin-HRP for 20 minutes, developed with 3,3′-diaminobenzidine (DAB) chromogen (Sigma, St. Louis, MO) for 3 minutes, and counterstained with hematoxylin. Wnt5a staining was scored as the intensity of staining in tumor cells (ignoring staining in surrounding non-tumor tissue) on a scale of 0 (no staining) to 3 (high intensity) by two independent observers. Cases with discrepant scores were re-evaluated jointly until agreement was reached.

### Cell culture

HTB-1™ (J82) and HTB-4™ (T24) urothelial carcinoma cell lines (ATCC, Manassas, VA) were cultured in Eagle’s Minimum Essential Medium and McCoy’s 5a Medium Modified, respectively, according to ATCC recommendations. The T24 cell line, characterized as non-tumorigenic, was derived from a poorly differentiated, high histological grade, recurrent tumor. The J82 cell line, with tumorigenic capacity, was derived from a poorly differentiated, high grade, invasive UC tumor [25]. The normal urothelial cell (NUC) line (Lifeline Cell Technology, Walkersville, MD) was cultured in ProstaLife™ Prostate Epithelial Cell Culture Medium according to the manufacturer’s recommendations. Control conditioned medium (cCM) was prepared using L cells (CRL-2648™, ATCC) grown in ATCC-formulated Dulbecco’s Modified Eagle’s medium plus 10% fetal bovine serum. Wnt5a-conditioned medium (Wnt5aCM) was prepared using L Wnt-5A cells (CRL-2814™, ATCC) grown in Dulbecco’s Modified Eagle’s medium with 4 mM L-glutamine provided by ATCC and supplemented with 0.6 mg/ml G418 and 10% fetal bovine serum. For each conditioned medium, the cells were split 1:10 in culture medium (without G418) in 10 cm petri dishes and grown for 4 days. The medium was subsequently removed and sterilized by filtration (first batch of conditioned medium). Fresh culture medium (without G418) was added and cultured for 3 more days. The medium was removed again and sterilized as above (second batch of conditioned medium). The first and second batches were mixed 1:1 and kept frozen until needed.

### Real time PCR

RNA was extracted from cells grown in 6-well plates using the RNeasy® Mini kit (Qiagen, Valencia, CA) following the manufacturer’s protocol. One microgram of RNA from each sample was reverse transcribed to cDNA using the High Capacity cDNA Reverse Transcription Kit (Applied Biosystems, Foster City, CA). Real-time quantitative PCR was performed in duplicate for each sample using a gene-specific Taqman probe, Taqman™ Gene Expression Master Mix, and a StepOnePlus™ Real-Time PCR System according to the manufacturer’s protocol (Applied Biosystems, Foster City, CA). Wnt5a (Hs00180103_m1) and the following six potential housekeeping genes were assayed: HPRT (Hs99999909), GAPDH (Hs99999905), ACTB (Hs99999903), B2M (Hs99999907), UBC (Hs00824723), and SDHA (Hs00188166). The most stable housekeeping genes were selected for normalization purposes using the Normfinder algorithm as outlined by Andersen et al. [26]. After normalization, data were presented as the fold change of Wnt5a RNA expression in each UC cell line relative to the level of expression observed in the NUC cells.

### Migration assay

Migration of T24 and J82 cells was qualitatively examined by scratch assay and then quantified using the Oris™ Cell Migration Assay kit (Platypus Technologies, LLC, Madison, WI) according to the manufacturer’s protocol. For the scratch assay, T24 and J82 cells were grown to confluence. The monolayers were washed with PBS, a cross was scratched into the surface of each monolayer, and then the cells were incubated with either regular medium (RM) in which each cell line normally grew, or cCM or Wnt5aCM, prepared as described above. Cellular migration was observed and visually recorded at 24 h intervals.

For the Oris Cell Migration Assay, a 96-well plate was populated with silicone stoppers and cells were added to each well. When the cells were confluent, the stoppers were removed and the clear area defined as the area before migration. The cells were then incubated under four different conditions: RM, RM supplemented with recombinant Wnt5a (R&D Systems) at 100 ng/ml (rWnt5a), cCM or Wnt5aCM. The cells were allowed to grow for 24 hours and then migration was stopped by fixation with 10% formalin. Each well was digitally imaged and traced, with the migration area defined as the percentage of the previously clear area now occupied by cells after 24 hours of growth. The measurements were performed using Image-Pro® Plus (Media Cybernetics, Inc., Silver Spring, MD).

### Statistical analysis

Logistic regression statistical analysis was performed to investigate the correlation between Wnt5a immunostaining and two variables, tumor histological grade and pathological stage. Cell migration was evaluated using t-tests to compare the effect of different treatments versus control. Statistical significance was tested at an alpha of 0.05. The software SPSS version 17 was used for data analysis (IBM SPSS, Armonk, NY).

## Results

### Wnt5a immunostaining increases with increasing histological grade and pathological stage of the tumor

Histological grade and pathological stage were determined in all 33 samples of human UC included in the study. The samples were diagnosed as noninvasive papillary UC of low grade (12 samples), noninvasive papillary UC of high grade (8 samples) or invasive UC (13 samples, all high grade). For pathological stage of the disease, 20 of 33 cases were in pTa, 7 in pT1, 5 in pT2, and one could not be determined.

**Figure 1:**
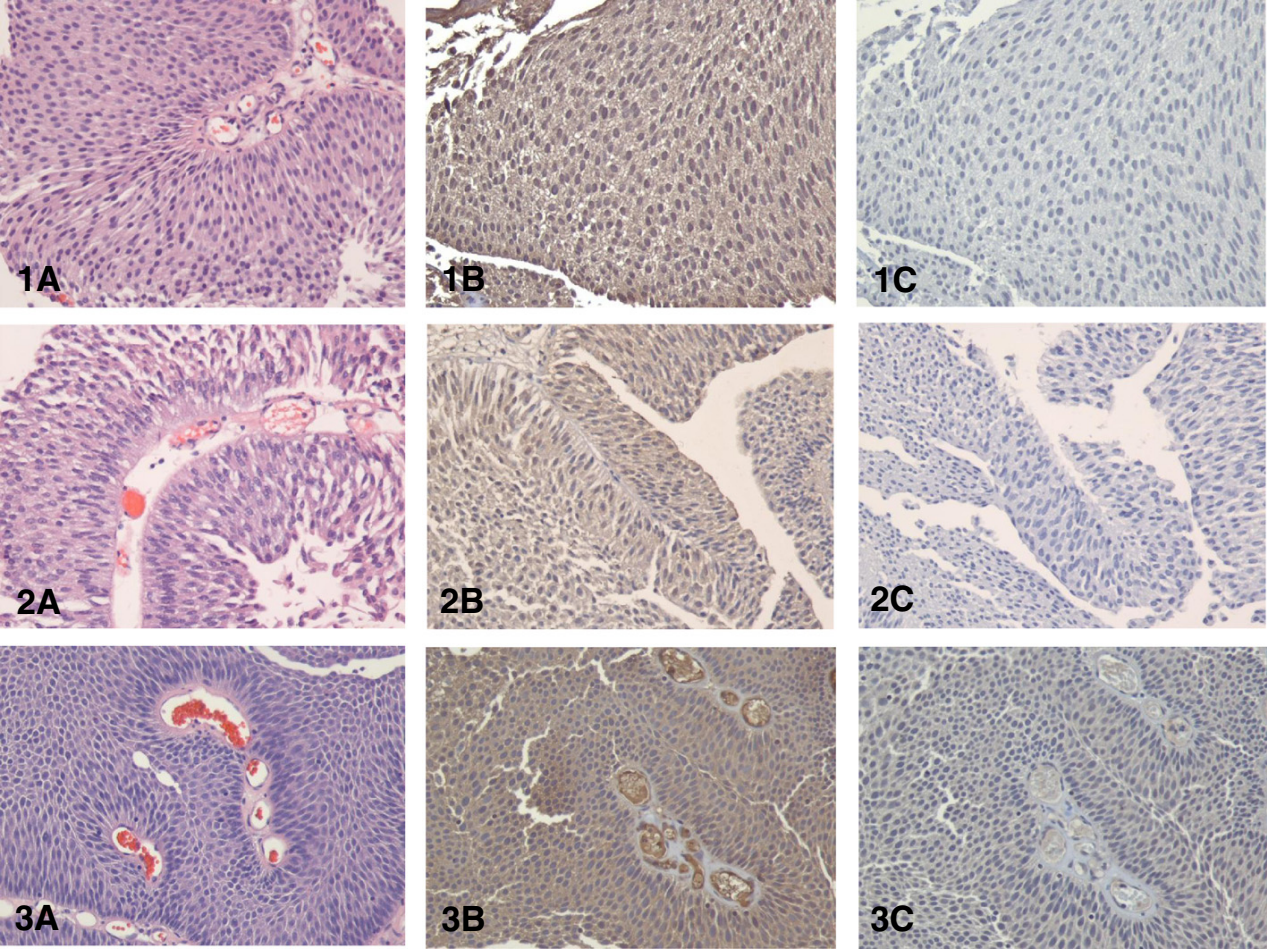
**Wnt5a expression in three cases of low grade, noninvasive, papillary urothelial carcinoma as assessed by IHC. (A)** H&E staining; **(B)** Wnt5a immuno-staining showing slight reactivity; **(C)** isotype control (all at × 200).

**Figure 2:**
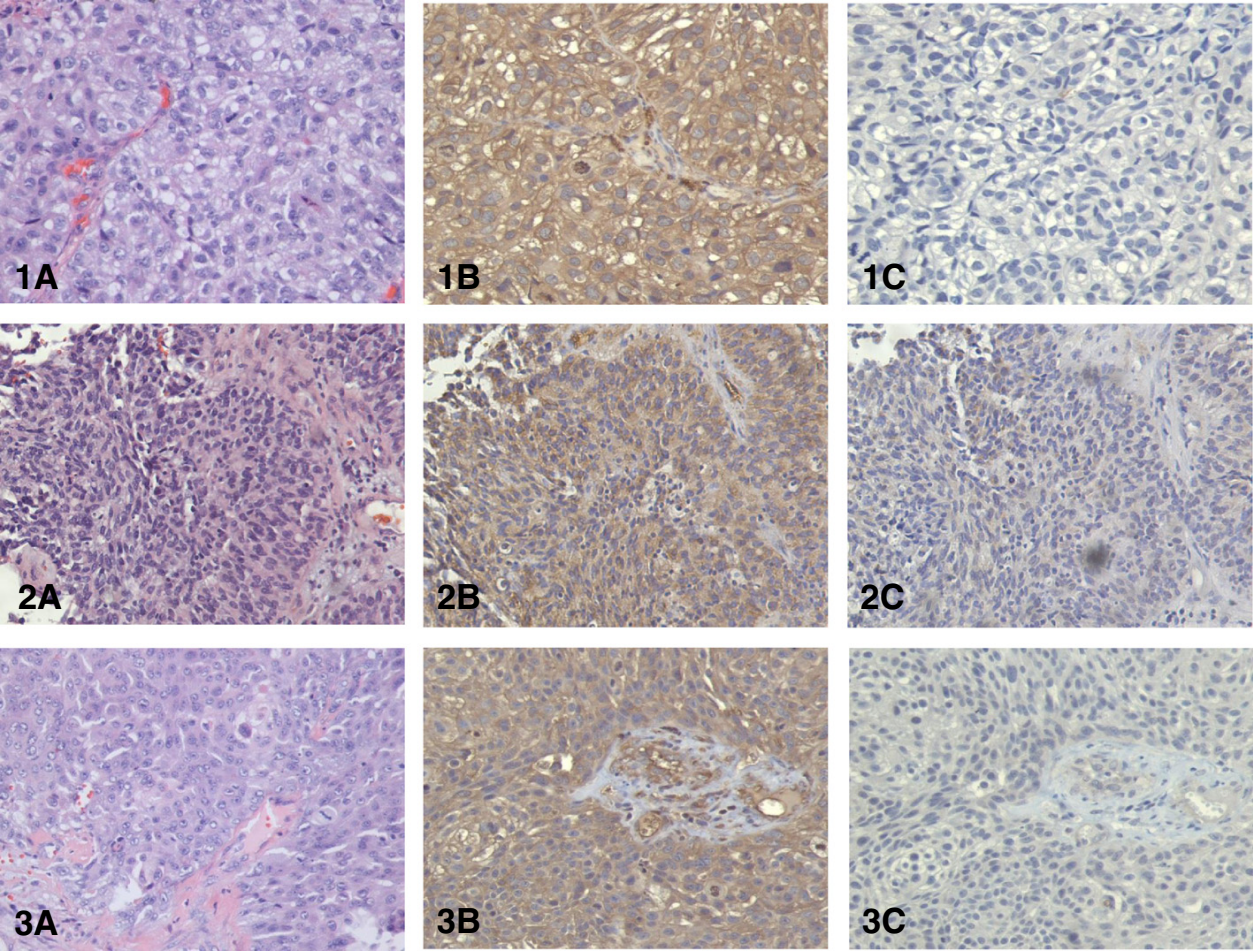
**Wnt5a expression in three cases of high grade urothelial carcinoma as assessed by IHC. (Top row)** Noninvasive urothelial carcinoma (pTa stage); **(middle row)** a case with lamina propria invasion (pT1 stage); **(bottom row)** a case with muscle invasion (pT2 stage); **(A)** H&E staining; **(B)** Wnt5a immuno-staining showing strong reactivity; **(C)**, isotype control (all at × 200).

**Figure 3:**
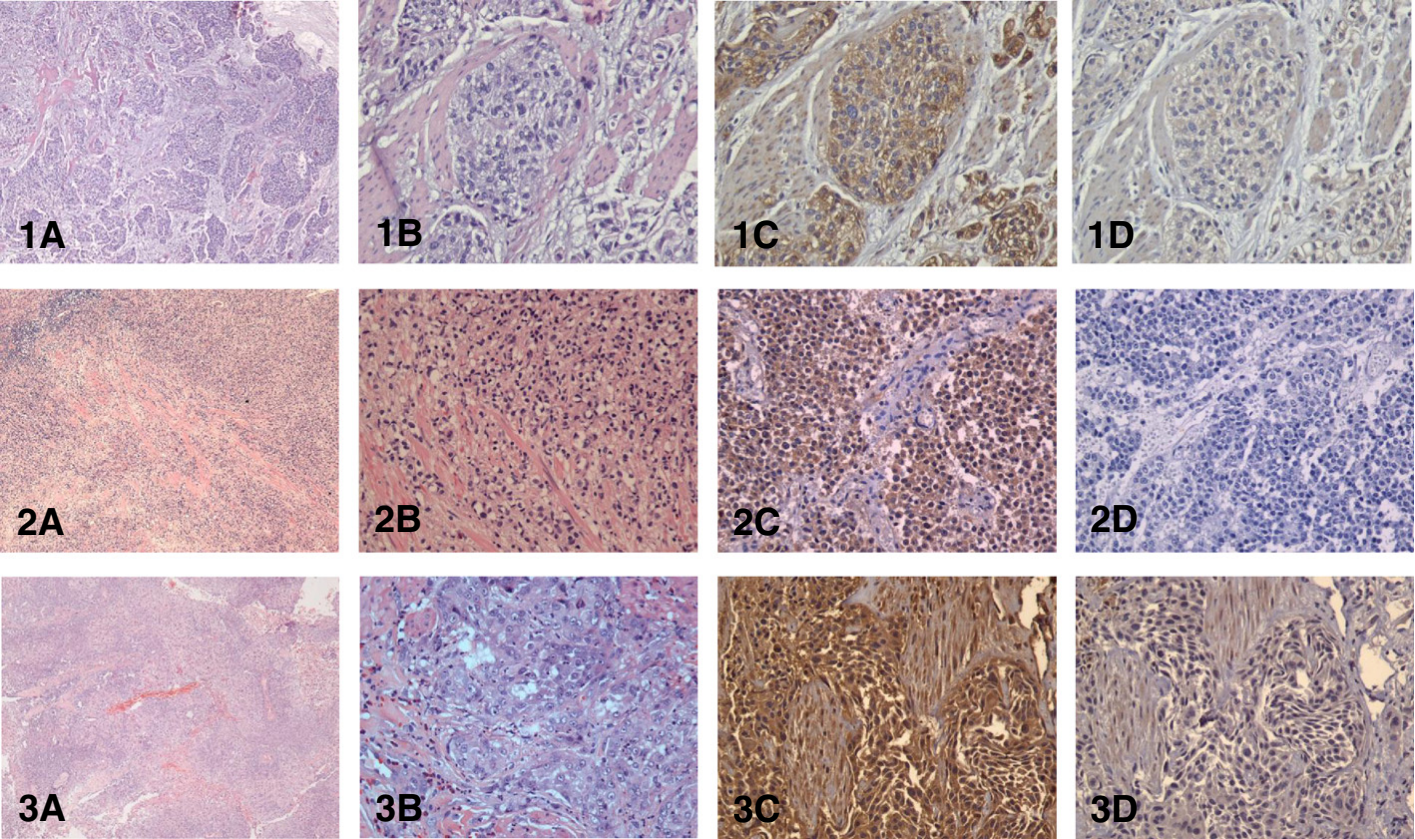
**Wnt5a expression in three cases of high grade, invasive urothelial neoplasms (pT2 stage) as assessed by IHC. (A)** H&E staining at low magnification shows deep invasion areas in the bladder wall (×40); **(B)** H&E staining (×200); **(C)** Wnt5a immune-staining showing strong reactivity (×200); **(D)** isotype control (×200).

Wnt5a protein was diffusely expressed by, and largely confined to, tumor cells in all 33 samples (Figures 1, 2 and 3). Specificity of Wnt5a staining was demonstrated by the lack of staining in the isotype controls. Intensity of Wnt5a staining positively correlated with the histological grade of the tumor (Table 1). Of the 12 samples classified as noninvasive papillary UC of low grade, six samples (50%) were 1+ for Wnt5a staining, four samples (33%) were 2+, and only two samples (17%) were 3+. In contrast, of the 21 samples classified as high grade, including noninvasive and invasive UC, 20 cases (95%) were 2+ or 3+ and only one case (5%) was scored as 1+. Staining intensity predicted a binary dependent variable of tumor grade (odds ratio = 1.29 [IC95: 1.03 ~ 1.60], p = 0.033); an increase in staining intensity was associated with an increased odds ratio (OR) of 1.29. This effect of staining intensity was not moderated by the stage (interaction between intensity and stage, p = 0.14), indicating that intensity predicted the tumor grade regardless of the stage. In fact, the staining intensity predicted the tumor grade at every stage (OR at stages 1–3 = 1.43, 2.00, 1.70; ps < 0.01).

**Table 1:** Correlation of Wnt5a staining intensity and histological grade of the tumors

		Wnt5a staining intensity^*^		
		**1+**	**2+**	**3+**
Histological grade	Low grade	6	4	2
	(n = 12)			
	High grade	1	10	10
	(n = 21)			

^*^ 1+ weak; 2+ moderate; 3+ strong.

Intensity of Wnt5a staining also positively correlated with the pathological stage of the tumor (Table 2). Of the cases with noninvasive disease (stage pTa), seven of 20 (35%) had a low score 1+; eight of 20 (40%) were 2+ and five of 20 (25%) scored 3+ for Wnt5a immonostaining. In contrast, of seven UC samples that had invasion into the lamina propria (stage pT1), five (71%) were 2+ and the remaining two (29%) were 3+. Further, of the five samples with invasion into detrusor muscle (stage pT2), one (20%) had a score of 2+ while the remaining four (80%) had a score of 3+. Statistical analysis showed that staining intensity predicts the tumor stage; the OR for the staining intensity effect was significant (OR = 4.37, p = 0.017). In other words, for one point increase in the staining score, the odds of the sample being at a more advanced stage increased by 4.37 [95% CI: 1.43-16.74].

**Table 2:** Correlation of Wnt5a staining intensity and pathological stage of the tumors

		Wnt5a staining intensity^*^		
		**1+**	**2+**	**3+**
Pathological stage^^^	pTa	7	8	5
	(n = 20)			
	pT1	0	5	2
	(n = 7)			
	pT2	0	1	4
	(n = 5)			
	N.D.	0	0	1
	(n = 1)			

^^^ pTa: non invasive; pT1: invasion to the lamina propria; pT2: invasion of the detrusor muscle; N.D.: could not be determined.

^*^ 1+ weak; 2+ moderate; 3+ strong.

Finally, correlation of stage, grade and Wnt5a staining of the UC samples was also assessed (Table 3). Not surprisingly, all 12 tumors showing invasive properties (pT1 or pT2) were also high grade and exhibited moderate to strong (2+ or 3+) Wnt5a staining. Interestingly, of the eight high grade, noninvasive tumors, only one (12.5%) had weak (1+) staining while seven (87.5%) had moderate to strong (2+ or 3+) Wnt5a staining.

**Table 3:** Correlation of stage, grade and Wnt5a staining intensity

**Stage**^**^**^	**Number of cases**	Staining intensity for low grade subset^*^	Staining intensity for high grade subset^*^
pTa	20	6 (1+)	1 (1+)
		4 (2+)	4 (2+)
		2 (3+)	3 (3+)
pT1	7	-----	0 (1+)
			5 (2+)
			2 (3+)
pT2	5	-----	0 (1+)
			1 (2+)
			4 (3+)
N.D.	1	-----	1 (3+)

^^^ pTa: non-invasive; pT1: invasion to the lamina propria; pT2: invasion of the detrusor muscle; N.D.: could not be determined.

^*^ 1+ weak; 2+ moderate; 3+ strong.

### Wnt5a mRNA expression differs widely between urothelial cell lines

**Figure 4:**
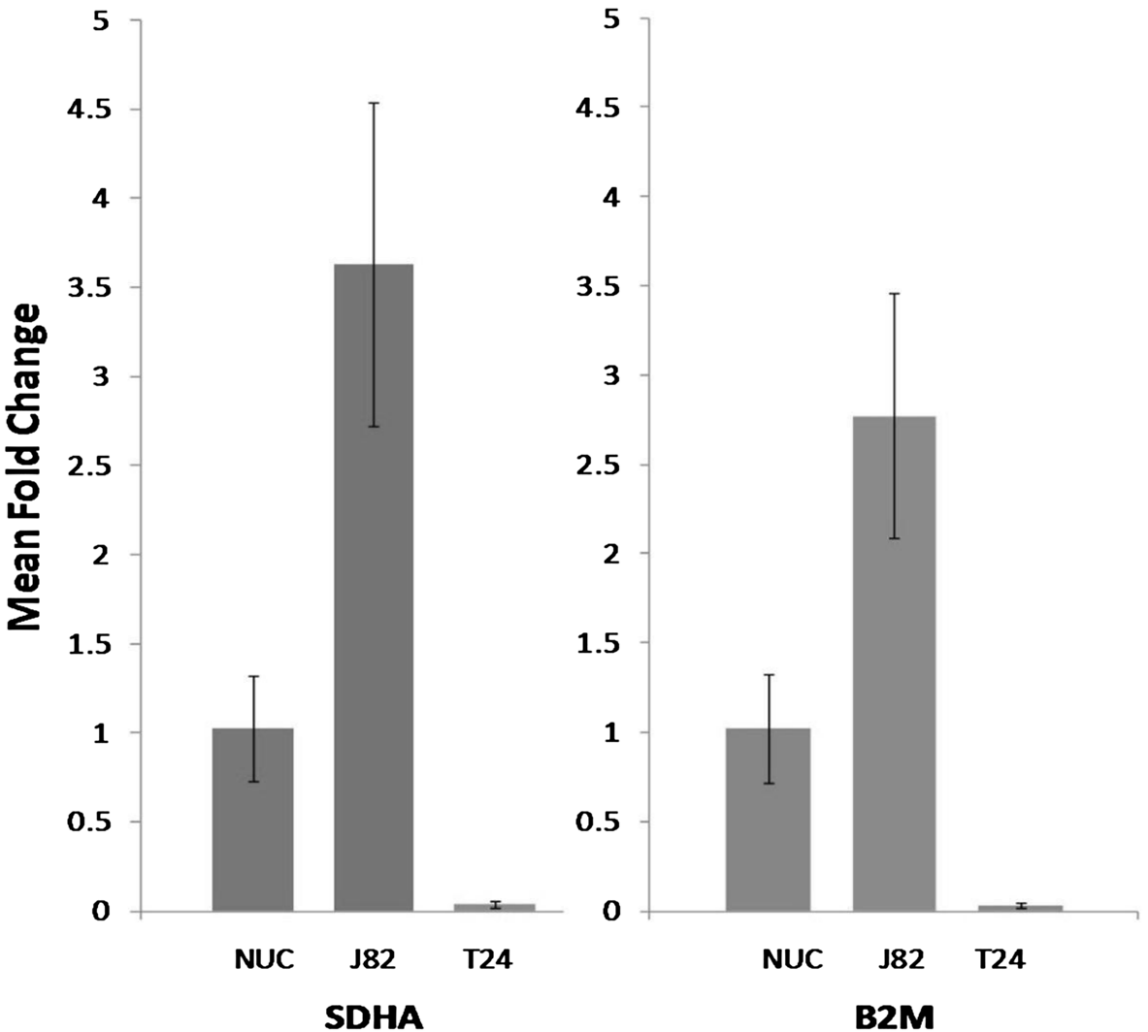
**Wnt5a mRNA expression in a normal urothelial cell line (NUC) and two urothelial carcinoma cell lines, J82 and T24, as assessed by real time RT/PCR.** The average fold change ± standard error (SE) for each cell line is shown in comparison to NUC after normalization with two different housekeeping genes, SDHA **(left)** and B2M **(right)**.

Wnt5a mRNA expression was compared among three cells lines: a normal urothelial cell line (NUC) and two different urothelial carcinoma cell lines (J82 and T24). Interestingly, in comparison to the normal urothelial cell line, Wnt5a mRNA expression was increased more than 3-fold in the J82 cell line but decreased 10-fold in the T24 cell line (Figure 4).

### Exogenously applied Wnt5a results in different migration rates of UC cell lines

**Figure 5:**
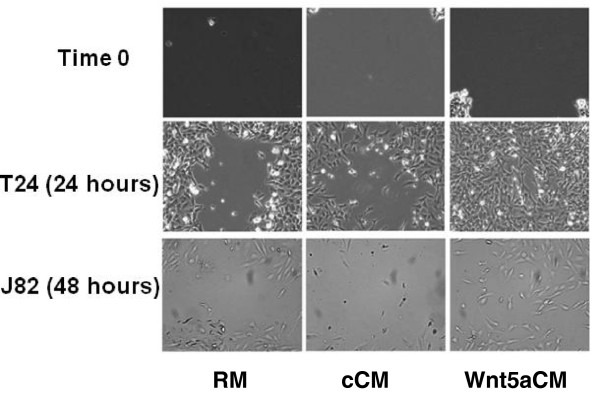
**Application of Wnt5a-conditioned medium increases migration of T24 and J82 cell lines in scratch assays.** Scratch assays are shown at time 0 (0 hours) and then 24 hours (T24) or 48 hours (J82) after the application of three different treatments: regular growth medium (RM), control-conditioned medium (cCM) and Wnt5a-conditioned medium (Wnt5aCM).

**Figure 6:**
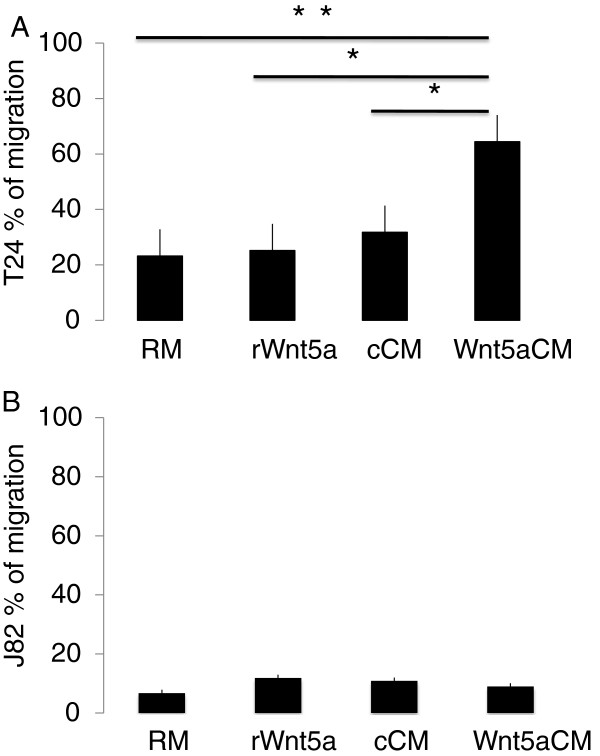
**Differing migration rates for T24 and J82 cell lines in response to exogenous Wnt5a.** Average migration area ± SE was determined using the Oris Cell Migration Assay kit 24 hr after application of regular medium (RM), regular medium supplemented with recombinant Wnt5a at 100 ng/ml (rWnt5a), control-conditioned medium (cCM), or Wnt5a-conditioned medium (Wnt5aCM). **(A)** Migration rate for the T24 cell line; **(B)** migration rate for the J82 cell line. * p = 0.05; ** p = 0.022.

Scratch assays showed that application of Wnt5a in the medium appears to increase the migration rates for both J82 and T24 UC cell lines, with more dramatic results for the faster growing T24 cell line (Figure 5). To validate and quantify these results, we repeated the experiment using an Oris Cell Migration Assay kit (Figure 6). In comparison to regular and control-conditioned media, application of Wnt5a-conditioned medium significantly increased migration of T24 cells. Addition of recombinant Wnt5a instead of conditioned medium had little effect on T24 migration. In contrast, addition of recombinant Wnt5a nearly doubled the migration rate for J82 cells, although the difference did not reach statistical significance. Addition of Wnt5a-conditioned medium had no significant effect.

## Discussion

Urinary bladder cancer is the fourth most frequent cancer in men in the world today [1-3]. Due to its great plasticity and biological variability with respect to multifocality, recurrence, and invasion comportment, it is difficult to predict the behavior of an initially noninvasive UC [2,3]. This feature makes the diagnosis of UC challenging, not only for its early detection, but also for accurate prediction of tumor recurrence and progression.

Multiple biomarkers used for other cancers have been described with potential diagnostic or prognostic value for UC as well, including p53, cytokeratin 20, E-cadherin, Ki67, CD44 and survivin [27,28]. Currently a combination of these immunohistochemical biomarkers assayed within panel is the best way to characterize subtypes and predict the behavior/aggresiveness of UC [28]. More recently, other promising biomarkers for UC have been reported. In a large retrospective exploratory study, expression of microRNA-100 was negatively associated with stage, recurrence, prognosis and death of patients with bladder cancer [29]. In another large study, the cell surface zinc-dependent metalloprotease CD10 was strongly correlated with tumor grade and stage and possibly associated with tumor progression in bladder cancer pathogenesis [30]. In contrast to initial studies correlating increased COX-2 expression and recurrence of urinary bladder cancer, a large study focused on non-muscle invasive bladder cancer found that lower levels of COX-2 and tumor-infiltrating lymphocytes were predictive of recurrence, suggesting the need of close follow-up and adjuvant therapy for these patients [31]. In the current study Wnt5a protein expression by malignant cells was observed in all 33 samples included in the study; however, intensities, and hence protein levels, varied, correlating with both grade and stage and suggesting that Wnt5a may also serve as a biomarker of UC. To prove the predictive nature of Wnt5a as a diagnostic or prognostic biomarker of UC, further investigation into the relationship between Wnt5a expression by the tumor and clinical progression of the disease in a large number of patients, is required.

Expression of Wnt5a by UC tumor cells may also be a predictor of an aggressive subgroup of UC. We found that weak Wnt5a expression was predominantly seen in low grade UC samples and all were in a noninvasive (pTa) stage. This suggests that low Wnt5a expression may be indicative of non-aggressive tumors. In contrast, moderate to strong Wnt5a expression was seen in all three pathological stages, but was usually associated with high grade, high stage tumors. Thus, moderate to strong expression of Wnt5a is likely to indicate an aggressive tumor or transformation to an invasive phenotype. In support of this hypothesis previous studies have reported the role of Wnt5a as a pro-angiogenic gene [32,33] and also the importance of the Wnt signaling pathway in epithelial-mesenchymal transition (EMT) of cancer [34]. In addition, Huang CL et al. reported the association of Wnt5a with tumor proliferation in non-small-cell lung cancer (NSCLC); the authors concluded that overexpression of Wnt5a could increase aggressiveness in NSCLC [35].

The present study also assessed Wnt5a mRNA expression in two UC cell lines, both from high grade, poorly differentiated tumors. Interestingly, the expression differed significantly between them, with one of the cancer cell lines expressing much higher levels and the other expressing much lower levels of Wnt5a mRNA in comparison to the normal urothelial cell line. This result is similar to the result published many years ago by Bui et al. in which they described different Wnt5a mRNA expression levels for different UC cell lines [36]. In that study the authors suggested that Wnt5a may modulate cell shape and cell migration [36].

In support of a role of Wnt5a in cell migration, Wnt5a has been associated with migration ability in melanoma [18]. Our group previously reported constitutive Wnt5a expression in human pancreatic cancer and malignant melanoma cell lines and suggested the role of Wnt5a in growth and migration in both of these cancer cell lines [19]. In this study we investigated migration response to exogenously applied Wnt5a in the two UC cell lines. We found that the T24 cell line, which has a low level of Wnt5a transcription, showed a significant increase in migration rate while J82, with high Wnt5a transcription, did not significantly respond to the application of Wnt5a. It is possible that J82, with high Wnt5a RNA, is already maximally saturated by Wnt5a and cannot respond further, while T24, with low Wnt5a RNA, is sub-maximally saturated and thus capable of responding to exogenous Wnt5a. Alternatively, it is possible that different cell lines have different responses to Wnt5a due to expression of different Wnt5a receptors. It is well known that the interaction between Wnt ligands and their receptors determine the signaling that will be activated, and multiple receptors may respond to Wnt5a signaling, each leading to activation of different downstream signaling pathways and effects [17,37,38]. Therefore, different responses might be associated with the presence of different receptors for Wnt5a ligand in each tumor cell line.

## Conclusion

In conclusion, our results support previous work suggesting that the Wnt5a signaling pathway plays a pathological role in UC. The correlation of Wnt5a protein expression in UC tissue sections with histological grade and pathological stage of the tumor suggests its possible use as diagnostic/prognostic tool for UC. Further studies to investigate association between Wnt5a expression, tumor behavior and clinical outcome are necessary to fully validate the utility of Wnt5a for early diagnosis/prognosis of UC. Further studies are also needed to clarify the underlying mechanisms connecting Wnt5a and different tumor behavior. These investigations promise to deliver predictability for a relatively unpredictable cancer.

## Competing interests

The authors declare that they have no competing interests.

## Authors’ contributions

RM conceived the project idea, provided funds, performed literature review, and drafted the majority of the manuscript. SC, DG and CB collected data, reviewed literature and drafted portions of the manuscript. KC analyzed and interpreted data and critically revised the manuscript. MN performed statistical analysis and reviewed the manuscript. SJ provided cases and reviewed the manuscript. All authors read and approved the final manuscript.
